# Loss of Neuronal Imp Contributes to Seizure Behavior through Syndecan Function

**DOI:** 10.1523/ENEURO.0545-24.2025

**Published:** 2025-05-02

**Authors:** Paula R. Roy, Nichole Link

**Affiliations:** Department of Neurobiology, University of Utah, Salt Lake City, Utah 84112

**Keywords:** *Drosophila*, neurogenetics, RNA-binding proteins, seizure

## Abstract

Seizures affect a large proportion of the global population and occur due to abnormal neuronal activity in the brain. Unfortunately, widespread genetic and phenotypic heterogeneity contributes to insufficient treatment options. It is critical to identify the genetic underpinnings of how seizures occur to better understand seizure disorders and improve therapeutic development. We used the *Drosophila melanogaster* model to identify that IGF-II mRNA-binding protein (Imp) is linked to the onset of this phenotype. Specific reduction of Imp in neurons causes seizures after mechanical stimulation. Importantly, gross motor behavior is unaffected, showing Imp loss does not affect general neuronal activity. Developmental loss of Imp is sufficient to cause seizures in adults; thus, Imp-modulated neuron development affects mature neuronal function. Since Imp is an RNA-binding protein, we sought to identify the mRNA target that Imp regulates in neurons to ensure proper neuronal activity after mechanical stress. We find that the Imp protein binds *Syndecan* (*Sdc*) mRNA, and the reduction of Sdc also causes mechanically induced seizures. Expression of Sdc in *Imp*-deficient neurons rescues seizure defects, showing that Sdc is sufficient to restore normal behavior after mechanical stress. We suggest that the Imp protein binds *Sdc* mRNA in neurons, and this functional interaction is important for normal neuronal biology and animal behavior in a mechanically induced seizure model. Since Imp and Sdc are conserved, our work highlights a neuronal-specific pathway that might contribute to seizure disorder when mutated in humans.

## Significance Statement

Imp is a widely studied RNA-binding protein in neural stem cell function, but surprisingly little is known about how it functions in postmitotic neurons in any model system. Here, we show that loss of neuronal Imp impairs mature neuronal function. Moreover, since RNA-binding proteins potentially regulate many targets, we provide a specific mechanism that illuminates how Imp could maintain normal neuronal function through downstream target *Syndecan*. Syndecan functions in cell adhesion, growth factor receptor activation, and intercellular signaling. Loss of Imp, and as a result, Syndecan, could cause defects in neuron migration, growth, or synapse morphology which could significantly impact neuronal function. On a broader level, our work highlights an important pathway to investigate human brain development and disease.

## Introduction

Seizure disorders are debilitating but common conditions. An estimated 10% of the human population will have at least one seizure in their life, and up to 3% of the global population experiences recurring spontaneous seizures defined as epilepsy (Shneker and Fountain, 2003). Despite their prevalence, phenotypic heterogeneity makes it challenging to identify seizure causality. Therefore, the development of patient-specific treatments can be difficult, but an understanding of the genetic pathways underlying seizure pathology may aid in the development of patient-directed therapies. Model organism research can greatly facilitate the identification of genetic-based seizure disorders (Tapia et al., 2021; Fischer et al., 2023). Genetic pathways can be studied in detail, mechanisms of pathogenesis are often uncovered, and drugs can be screened in a patient-specific manner to identify candidate therapeutics.

A class of genes with a growing connection to syndromes involving seizures are RNA-binding proteins (Schieweck et al., 2021). Therefore, the contribution of RNA-binding proteins in neurodevelopment and neurological disease is an emerging interest in the fields of genetics and neuroscience. RNA-binding proteins spatially and temporally regulate gene expression to coordinate and modify dynamic cellular networks like those found in the brain (Prashad and Gopal, 2021; Parra and Johnston, 2022). Insulin growth factor 2 binding proteins (IGF2BPs) are mRNA-binding proteins that post-transcriptionally regulate mRNA by influencing localization, transport, translation, and stability (Nielsen et al., 2001; Yaniv and Yisraeli, 2002). Vertebrates have three IGF2BP paralogs (1–3) that are expressed during development, with high expression in neuronal cells (Conway et al., 2016; Degrauwe et al., 2016). Though there is no current functional validation of IGF2BPs to specific neuropathologies, *IGF2BP3* is a primary microcephaly candidate (Duerinckx et al., 2021), and all IGF2BPs are associated with multiple targets connected to autism spectrum disorder (Pintacuda et al., 2023). There is also much model organism evidence to support the critical role of *IGF2BP*s in brain development. *IGF2BP1* influences pluripotent stem cell adhesion and survival (Conway et al., 2016; Degrauwe et al., 2016), neural stem cell maintenance (Nishino et al., 2013), hippocampal dendrite arborization (Perycz et al., 2011), and axon guidance and outgrowth (Gaynes et al., 2015; Lepelletier et al., 2017). IGF2BP2 localizes in dendrites (Tiruchinapalli et al., 2003) and in neural precursors, where it mediates neurogenic and astrocyte differentiation potential (Fujii et al., 2013). The single ortholog in *Drosophila* (*Imp*) is structurally conserved with vertebrate IGF2BPs (Hansen et al., 2015) and is a well-documented temporal factor in neural stem cells, where it is expressed in a high to low gradient starting in embryogenesis (Adolph et al., 2009). Descending Imp levels through the larval stage defines stem cell growth and proliferation (Yang et al., 2017; Samuels et al., 2020), neuronal fate and diversity (Ren et al., 2017; Yang et al., 2017; Munroe et al., 2022), and stem cell decommissioning (Yang et al., 2017; Samuels et al., 2020). Specifically, Imp promotes *Chinmo* translation and consequently represses *mamo* transcription (Liu et al., 2015) to control the specification of early and late-born neurons (Liu et al., 2015). Imp also controls the growth and proliferation of neural stem cells by stabilizing *Myc* transcripts (Samuels et al., 2020) and localizes to axons suspected to regulate regrowth during axon remodeling via *prolifin* mRNA transport and localization (Medioni et al., 2014). In both stem cells and intermediate neural progenitors, *Imp* facilitates proper neuropil targeting to the major learning and memory centers in the adult brain (Munroe and Doe, 2023) and is required for proper development of adult olfactory navigation circuitry (Hamid et al., 2023). Together, these studies show that *Imp* function in neural stem cells during development affects both neuronal morphology (Medioni et al., 2014; Vijayakumar et al., 2019) and neuronal fate (Liu et al., 2015; Munroe and Doe, 2023; Guan et al., 2024) in adults.

While previous work highlights the critical role that *IGF2BP*s/*Imp* have on neurodevelopment, research to date has primarily focused on neural stem cells or intermediate neural progenitors. Furthermore, the functional consequences of developmental Imp loss are vastly understudied. This leads us to ask: how does Imp regulate neurons both developmentally and functionally, and how does disruption of *Imp* in these neurons correlate to human neuropathologies? We hypothesize that Imp is required in neurons themselves for normal neuronal function, and to test this hypothesis, we leveraged the *Drosophila* model system to spatially and temporally knock down *Imp* expression. We found that neuronal Imp is required during development to ensure proper neuronal function that is specific to seizure behavior. We also found a smaller role for Imp independent of development, because the loss of Imp in adulthood caused seizures. We also identified that loss of Imp target, *Syndecan* (*Sdc)*, caused seizures and showed with rescue assays that Sdc functionally interacts with Imp in seizure behavior. Together, we show that neuronal Imp is essential in developing neurons for adult neuronal function and that the interaction between Imp and *Sdc* modulates seizure behavior.

## Materials and Methods

### *Drosophila melanogaster* strains and culture

The following lines were used in this study with genotypes separated by commas: w[*]; *P{w[ + mW.hs] = GawB}insc[Mz1407]* (RRID: BDSC_8751), *y[1] w[1118]; P{y[ + t7.7] w[ + mC] = nSyb-GAL4.P}attP2* (RRID: BDSC_51941), y[1]sc[*] v[1] sev [21]; P{y[ + t7.7] v[ + t1.8] = VALIUM22-EGFP.RNAi.1}attP40 (RRID: BDSC_41557), y[1]sc[*] v[1] sev [21]; P{y[ + t7.7] v[ + t1.8] = TRIP.HMC03794}attP40 (RRID: BDSC_55645), *P{KK108799}VIE-260B* (RRID:Flybase_FBst0479142), w[*]; P{w[ + mC] = UAS-Sdc.J}3 (RRID: BDSC_8564), P{UAS-t, y[1]w[*] Mi{PT-GFSTF.2}Imp{M105901-GFSTF.2 (RRID: BDSC_60237), y[1] w[*]; Mi{PT-GFSTF.0}Sdc[MI10787-GFSTF.0]/Cyo (RRID: BDSC_66373).

All lines were maintained at either 25 or 18°C on Archon Scientific glucose medium supplemented with dry yeast in wide plastic vials. All incubators were kept on a 12 h light/dark cycle.

### Behavior assays

#### Standard behavior conditions

All flies were aged to 14 d. Crosses were set by placing 10 virgin GAL4 driver females into a single vial with five UAS males. A minimum of three replicate vials per experimental group were set, and for each individual, the vial identification was tracked to ensure that there were no cross/vial effects. Carbon dioxide can acutely alter behavior (Bartholomew et al., 2015), so flies were only anesthetized with carbon dioxide when collected as virgins and later moved into test vials using mouth pipetting. After transferring to a new vial, animals were left to acclimate for 20 min at room temperature. All behavior trials were performed within 4 h of lights on. Females exhibited a more consistent phenotype in *Imp* pilot seizure assays (Extended Data Fig. 1-1), so only females were used for data collection. All trials were recorded using either an iPhone 13 mini or a Panasonic DMC-G85 camera with an Olympus ED 60 mm f2.8 macro lens.

#### Seizure assays

Female progeny from each experimental genotype were matched to the same driver control knockdown (*eGFP*) and aged at 29°C. Standard bang sensitivity assays, specifically vortex assays, were performed to assess seizure behavior (Fischer et al., 2023) where vials were vortexed for 10 s and placed on the bench at eye level to easily record flies. The proportion of flies seizing and time to recovery were recorded for each vial. Seizing was defined by supine paralysis, spastic movement, and/or inability to walk with excessive tremoring. Recovery was defined as walking normally in the correct orientation without abdominal or wing spasms and/or exhibiting normal geotaxis behavior without abdominal/wing spasms or falling. Some flies climbed very quickly after flipping over and did not walk along the bottom of the vial. Trials were ended once all flies had recovered fully or until 60 s.

#### Developmental/functional assays

*Imp* RNAi crosses were set, and progeny developed (egg, larval, and pupal stages, then up to 4 h post-eclosion), at either 18°C (low GAL4 activity temperature) or 29°C (high GAL4 activity temperature). Within 4 h of eclosion, adults were collected and moved into fresh vials and either left at their developmental temperature or moved to the inverse temperature to age for 14 d. On the assay day, aged flies were aspirated into empty vials in sets of up to 15 and acclimated for 20 min at room temperature. Flies were analyzed for seizure using the standard bang assay described in the initial knockdown assays.

#### Rescue assays

Female progeny were collected within 8 h of eclosion and aged to 14 d at 29°C. On seizure assay days, aged flies were aspirated into empty vials in sets of up to 10 and acclimated for 20 min at room temperature. Flies were analyzed for seizure using the standard bang assay described in the initial seizure assays.

#### Forced-climbing assays

Female progeny were developed and aged to 14 d at 29°C. On the day of testing, aged female flies were aspirated into empty vials marked with 1 cm lines in sets of up to 10 and acclimated for 20 min at room temperature. Vials were banged on the benchtop three times to ensure that all flies were at the vial bottom and were allowed 60 s to climb to their maximum distance (up to 9 cm). The maximum distance climbed by each fly was recorded.

### Quantitative PCR

Brains from late third-instar larvae (three biological replicates of 10 brains for each genotype) were dissected in RNAse-free PBS, rinsed in RNAse-free water, flash frozen on dry ice, and stored at −80°C. Total RNA was isolated using standard TRIzol extraction (Green and Sambrook, 2020), synthesized into cDNA (iScript cDNA synthesis, Bio-Rad #1708890**),** and stored at −20°C. Primer sequences for *Imp*: (F) CGTCACGCGCTGCAATTC (R) GCTTCATGTGTGGCACGGAC. Primer sequences for *Rp49/RpL32*: (F) ATGACCATCCGCCCAGCATACA (R) CGTAACCGATGTTGGGCATCAGATACT. Primers were optimized with nonquanitative fast-run PCR (PowerTrack SYBR Green Master Mix, Applied Biosystems #46012) with a temperature gradient (55–65°C) to determine the best annealing temperature. Quantitative PCR was performed on a Life Technologies QuantStudio 12 K Flex instrument at the University of Utah Genomics Core Facility. Rp49/RpL32 was used to normalize *Imp* knockdown levels.

### RNA immunoprecipitation

Forty *yw* (control) and y[1]w[*] Mi{PT-GFSTF.2}Imp{M105901-GFSTF.2 (RRID: BDSC_60237) larvae were collected, fixed in 0.1% paraformaldehyde (EM grade, Electron Microscopy Sciences #15710) in 1× phosphate-buffered saline (PBS) plus 0.3% Triton X-100 for 10 min. From here, all steps were carried out in an RNAse-free manner with RNAse inhibitors if needed. Larvae were rinsed with 0.125 M glycine to stop fixation, rinsed in PBS, and transferred to Chaps cell extract buffer (Cell Signaling Technology #9852) with Pierce phosphatase and protease inhibitors (Thermo Fisher Scientific #A32961) or Halt protease and phosphatase inhibitor cocktail (Thermo Fisher Scientific #78440). Animals were ground with a motorized pestle, frozen at −80°C, thawed on ice, and vortexed three times. Debris was pelleted by centrifuging for 10 min at 4°C. Supernatant was transferred to a new tube. Fifty microliters were saved for RNA and protein input samples. A Chromotek GFP-Trap Agarose kit was used to immunoprecipitate Imp-GFP and wash the sample. Fifty microliters of lysate flow were saved for RNA and protein analysis. One-third of immunoprecipitate was saved for protein analysis. To disassociate RNA from Imp-GFP/beads, GFP agarose was resuspended in 100 µl lysis buffer (from the Chromotek kit) with 30 µg proteinase K and incubated for 30 min at 55°C in a thermoshaker with agitation. RNA was extracted using TRIzol (Thermo Fisher Scientific #15596026). Six hundred microliters of TRIzol were added, mixed by pipetting up and down, and incubated at room temperature for 5 min. One hundred and fifty microliters of chloroform were added, tubes were shaken vigorously for 15 s, and samples were incubated for 10 min at room temperature. Tubes were centrifuged for 15 min at 4°C, and the aqueous phase was removed and saved. Subsequently, 1.5 µl of GlycoBlue and 500 µl of isopropanol were added and tubes were vortexed for 10 s. Samples were incubated overnight at −20°C and centrifuged for 15 min at 4°C for precipitation. RNA pellets were washed with 70% ethanol, and dried pellets were resuspended in 20 µl of RNAse-free water. cDNA was generated using a Bio-Rad iScript cDNA synthesis kit (Bio-Rad #1708890). Primers specific for Sdc were used for PCR (forward: TCCTACGGCCATAGCCATTAT; reverse: CGGTAGAAGTAATTCCGCCAGA).

### Larval dissection and immunohistochemistry

Brains from late third-instar larvae (identified by gut clearing and spiracle protrusion) were dissected in cold phosphate-buffered saline (PBS), fixed in 4% paraformaldehyde in PBS plus 0.3% Triton X-200 (PBST) for 20 min, then washed in PBST for 5 min three times before blocking in PBST plus 1% bovine serum (PBSTB) for 30 min two times. Brains were blocked a third time in PBSTB plus 5% normal donkey serum (NDS) for 30 min. Brains were incubated in primary antibodies diluted in PBSTB for 3 d at 4°C, washed with PBSTB for 20 min three times, and then incubated in secondary for 2 d at 4°C. Brains were washed in PBST four times for 15 min, adding DAPI into the second wash, and mounted in SlowFade gold on microscope slides with spacers made from a single layer of double-sided tape.

We used the following antibodies in this study: rabbit anti-GFP (1:1,000, Invitrogen A-11122, RRID: AB_221569), rat anti-Imp (1:200, a gift from Dr. Claude Desplan, New York University), mouse-anti ElaV (1:100, Developmental Studies Hybridoma Bank 9F8A9, RRID: AB_2314364), donkey anti-rabbit 488 (1:500, Jackson ImmunoResearch 711-545-152, RRID: AB_2313584), donkey anti-rat fluorophore 647 (1:500, Jackson ImmunoResearch 712-605-153, RRID: AB_2340694), and donkey anti-mouse fluorophore rhodamine red-X secondary (1:500, Jackson ImmunoResearch 715-295-151, RRID: AB_2340832).

Brains were imaged on a Zeiss LSM980 confocal microscope using Airyscan. Single 2 um slices through the middle of the brain were imaged with a 63× oil objective.

### Statistics

For seizure assays, the total time to recovery was analyzed between control and treatment groups using a Mann–Whitney *U* test for single comparisons and a Kruskall–Wallis test for multiple comparisons. For motor assays, the maximum distance climbed was analyzed between control and treatment groups with a Mann–Whitney *U* single-comparison test. We chose nonparametric tests because the variation in behavior within genotypes follows non-normal distributions. All statistical analyses were performed using GraphPad Prism version 10.0 for Mac OS, GraphPad Software, Boston, MA, USA, www.graphpadprism.com. Minimum *N* size was determined using seizure data from our pilot neural stem cell and postmitotic knockdown experiments to perform an a priori sample size computation in G*Power (Faul et al., 2007) using *α* = 0.5 and a power set to 0.80. The threshold for sufficient power was *N* = 40. The power of each test was determined using post hoc analysis in G*Power (Faul et al., 2007) using *α* = 0.5 and power set to 0.80 (Table 1).

**Table 1.** Power analysis results for all behavioral assays determined post hoc in G*Power

Experiment	Corresponding figure	Data structure	Statistical test	Power
Seizure assay cell-specific *Imp* knockdown	Figure 1*A*	Abnormal, unequal variance	Kruskal–Wallis	0.9999965
Forced-climbing assay *Imp* knockdown	Figure 1*C*	Abnormal, unequal variance	Mann–Whitney *U*	0.9830928
Seizure assay staging *Imp* knockdown	Figure 2*A*	Abnormal, unequal variance	Kruskal–Wallis	1.0000000
Seizure assay *Sdc* knockdown	Figure 3*A*	Abnormal, unequal variance	Mann–Whitney *U*	0.9999187
Forced-climbing assay Sdc knockdown	Figure 3*B*	Abnormal, unequal variance	Mann–Whitney *U*	0.8732388
Seizure assay Sdc rescue	Figure 3*I*	Abnormal, unequal variance	Kruskal–Wallis	1.0000000

## Results

### Loss of Imp causes seizures with normal gross motor function

**Figure 1.**
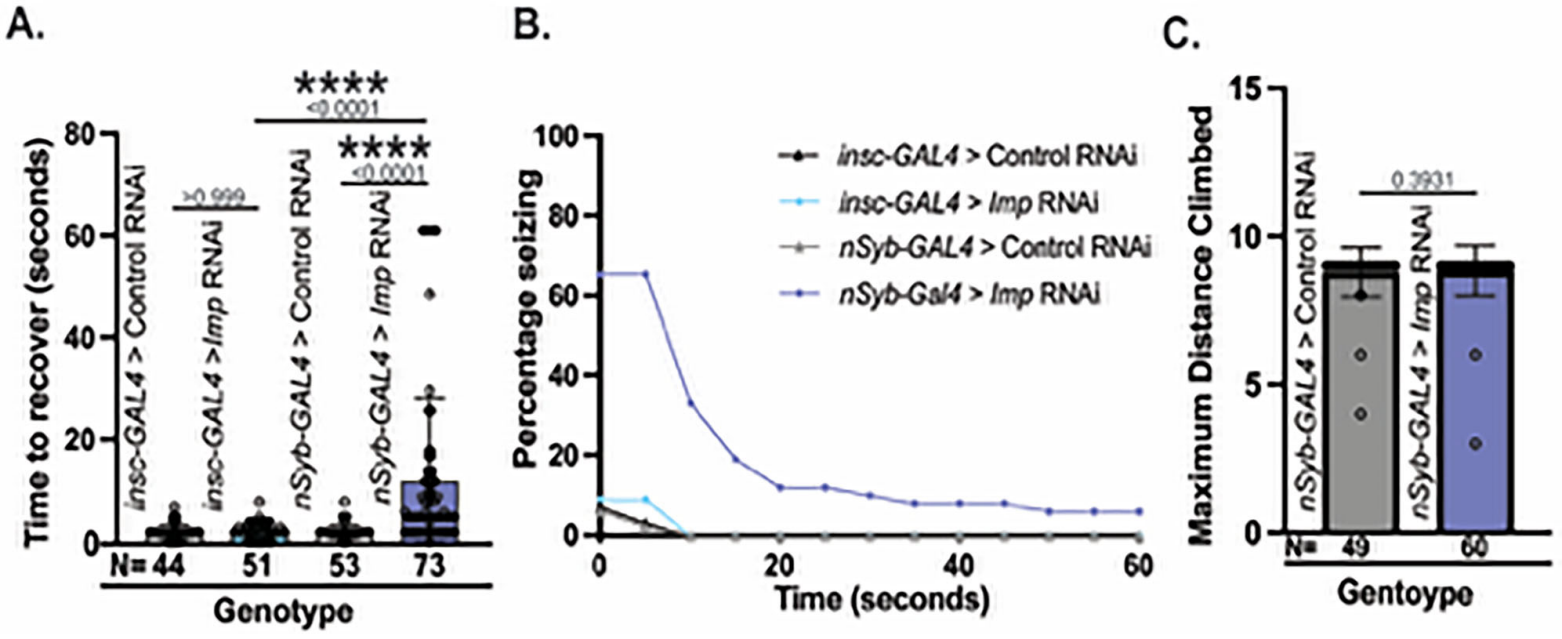
*Imp* knockdown causes higher occurrence and longer duration of seizures with normal gross motor function. ***A***, Seizure behavior reported as average time to recover after vortexing for *eGFP* RNAi control (*VALIUM22*-*EGFP.shRNAI.1*) and *Imp* RNAi (*TRIP.HMC03794*) expressed using neural stem cell driver *inscuteable*-*GAL4* (*insc-GAL4*) or pan-neuronal driver *neuronal synaptobrevin-GAL4* (*nSyb-GAL4*). Individual points represent each fly, whiskers represent 95% confidence intervals, and bar heights equal the mean. Kruskal–Wallis test determined the significance between all conditions. Relevant comparisons reported (*p* < 0.05, ***p < 0.01, ***p < 0.001,****p < 0.0001, ns = *p* > 0.05). *N*, number of total animals. ***B***, Percentage of flies seizing reported in ***A*** observed at 5 s intervals across the entire 60 s trial. *N*s are the same as in ***A***. Seizing is defined by supine paralysis, spastic movement, and/or inability to walk with excessive tremoring. Behavioral assays were performed on Day 14. ***C***, Maximum distance climbed (cm) in 30 s for *eGFP* RNAi control and *Imp* RNAi expressed in *nSyb-GAL4*. Individual points represent individual flies, whiskers represent 95% confidence intervals, and bar heights equal the mean. *p*-values are reported above each bar. Mann–Whitney *U* determined significance (*p* < 0.05, ***p < 0.01, ***p < 0.001,****p < 0.0001, ns = *p* > 0.05). *N*, number of total animals. All animals were female because their seizure phenotypes were more consistent (Extended Data Fig. 1-1).

Imp has been highly characterized in neural stem cells where it functions as an important temporal factor to influence the type of neurons produced at different developmental time points (Liu et al., 2015). However, it is unknown how loss of neuronal *Imp* might affect brain function independent from its role in stem cells. To test Imp's neuronal function and resulting behavior outputs, we assessed seizure phenotypes using vortex assays. We reduced *Imp* using in vivo RNA interference (RNAi) in two primary cell types: neural stem cells and postmitotic neurons. Loss of *Imp* in both cell types resulted in some animals seizing, but only neuronal knockdown animals seized significantly more than controls [Fig. 1*A*, Kruskal–Wallis, *p *> 0.999 (neural stem cells) and *p *< 0.0001 (neurons)]. Loss in postmitotic neurons caused both a higher frequency of seizures and a trend of longer recovery from seizures (Fig. 1*B*). The average length of seizure was 9.67 s in animals with *Imp* knockdown in neural stem cells. However, *Imp* knockdown in neurons caused seizures for an average of 22.38 s, correlating neuronal *Imp* loss to a more severe phenotype (Kuebler and Tanouye, 2002). The difference in recovery time between stem cell and neuronal *Imp* knockdown was significantly different (Kruskal–Wallis, *p *< 0.0001, Fig. 1*A*). All neural stem cell knockdown animals recovered in under 10 s, and once animals recovered, they did not experience additional seizures (Movies 1 and 2). Neuronal knockdown animals had a more conspicuous phenotype. Noticeable “tonic–clonic” phases with paralysis were interrupted by multiple bouts of spasms during the recovery phase (Fischer et al., 2023). Individuals experiencing multiple episodes demonstrated periods of flips, brief walks, and a return to supine position, which corresponded to “clonus-like” activity (Movies 3 and 4). Neuronal knockdown produced a robust and consistent seizure phenotype, supporting the idea that neuronal Imp regulates neuron function in the context of seizure behavior. Furthermore, this function was not dependent on *Imp*'s role in neural stem cells. As a result, the remainder of the analyses were performed only on neuronal knockdown animals.

10.1523/ENEURO.0545-24.2025.f1-1 Figure 1-1 *Imp* knockdown has a stronger effect on females. Seizure behavior reported as average time to recover after vortexing for *eGFP* RNAi (VALIUM22-*EGFP.shRNAI.1*) Control and *Imp* RNAi (*TRIP.HMC03794*) expressed using pan-neuronal driver *neuronal synaptobrevin-GAL4* (*nSyb-GAL4*). Individual points represent each fly, whiskers represent 95% confidence intervals, and bar heights equal the mean. Kruskal-Wallis test determined significance between all conditions. Relevant comparisons reported (p<0.05, ***p<0.01, ***p<0.001,****p<0.0001, ns=p>0.05). N= number of total animals. Download Figure 1-1, TIF file.

Flies have a natural tendency to climb that is inhibited if animals are experiencing gross motor defects (Cao et al., 2017). Climbing deficiencies in *Imp* knockdown animals could suggest a general motor deficit rather than a specific functional defect. To differentiate between gross deficits and acute, induced functional disruptions, we used forced-climbing assays. Aged flies were placed into vials, banged to the bottom, and allowed to climb to a maximum distance (9 mm) for 60 s to determine if animals had any broad motor deficits that prevented climbing. We found that loss of neuronal *Imp* did not affect climbing ability (Mann–Whitney *U*, *p *= 0.3931, Fig. 1*C*), suggesting that the observed mechanically induced seizure phenotypes were not due to gross motor problems. Therefore, neuronal Imp functions in seizures but not general motor function.

### Developmental Imp loss is the primary contributor to seizure behavior

**Figure 2.**
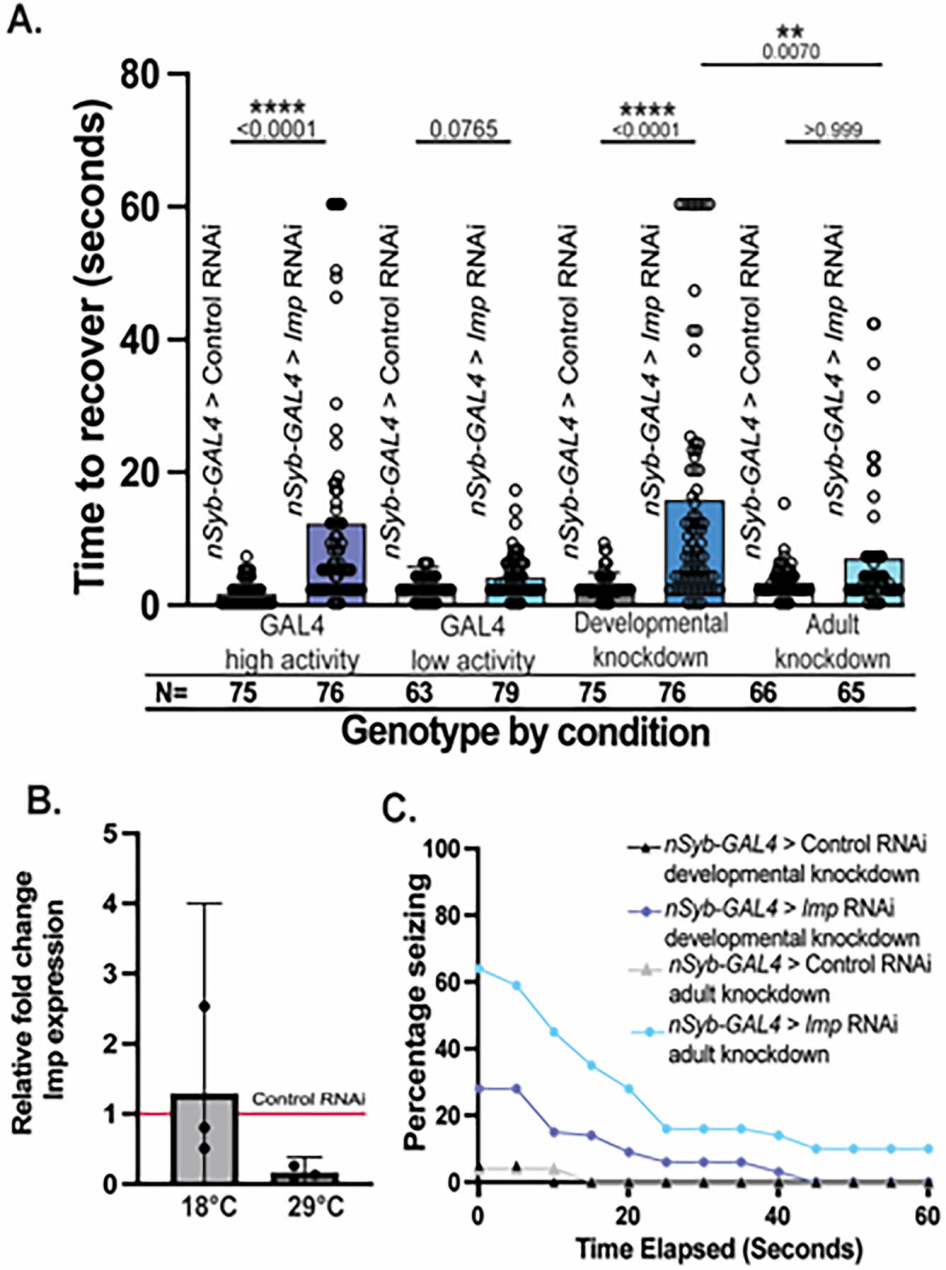
Developmental loss of neuronal Imp is the primary source of seizure behavior. ***A***, Average time to recover for flies exhibiting seizure behavior for neuronal (*nSyb-GAL4*) *eGFP* RNAi (control) and neuronal *Imp* RNAi. High GAL4 activity animals are raised at 29°C and low GAL4 activity animals are raised at 18°C. Results demonstrate that *nSyb-GAL4* RNAi is temperature sensitive. Developmental knockdown animals are raised at 29°C and moved to 18°C within 1 d of adulthood. Adult knockdown animals developed at 18°C and shifted to 29°C within 4 h of adulthood. Behavioral assays were performed on Day 14. Individual points represent individual flies, whiskers represent 95% confidence intervals, and bar heights equal the mean. *p*-values are reported above each bar. Kruskal–Wallis determined significance between all conditions. *N*, number of total animals. Relevant comparisons reported (*p* < 0.05, ***p < 0.01, ***p < 0.001,****p < 0.0001, ns = *p* > 0.05). Developmental knockdown of *Imp* is sufficient to recapitulate the seizure phenotype. ***B***, Fold change in *Imp* expression in third-instar larval brains developed at 18 and 29°C. Individual points represent a biological replicate group of 10 larval brains, whiskers represent 95% confidence intervals, and bar heights equal the mean. ***C***, Percentage of flies presented in ***A***, seizing observed at 5 s intervals across the entire 60 s trial.

RNA-binding proteins are broadly acting genes affecting both cell development and mature cell function (Prashad and Gopal, 2021; Chan et al., 2022; Parra and Johnston, 2022). To determine when *Imp* loss results in seizures, we used the GAL4-UAS system to temporally remove *Imp* in neurons. Some *GAL4-UAS* combinations have a temperature-sensitive effect where strong activity occurs at high temperatures (high activity, 29°C) and little-to-no activity occurs at low temperatures (low activity, 18°C). We verified phenotype-specific temperature sensitivity by comparing recovery times in vortex assays between control and *Imp* knockdown animals at high activity and low activity conditions (Fig. 2*A*). We found a significantly longer recovery time using the high activity conditions (29°C, Kruskal–Wallis *p *< 0.0001) but not the low activity conditions (18°C, *p *= 0.0765), showing that *nSyb-GAL4* combined with our *Imp* RNAi construct has a temperature-sensitive phenotype that can be used to modulate the severity of *Imp* knockdown. Additionally, we validated *Imp* knockdown at both high and low activity temperatures using quantitative PCR (Fig. 2*B*) and found an average decrease in *Imp* expression by 84% at 29°C but no decrease in expression at 18°C. We used this temperature-sensitive phenotype to disentangle Imp function during development versus in the adult (Fig. 2*A*). We placed developing animals in the low activity condition (18°C) and moved adults to the high activity condition (29°C) upon eclosion. Our schedule was designed to isolate *Imp* knockdown to adulthood only. Seizure activity increased in animals with *Imp* knockdown only during adulthood, but it was not significantly different from its eGFP RNAi control match (Kruskal–Wallis, *p *> 0.9999). Next, we placed developing animals in the high activity condition (29°C) during development and upon eclosion moved adults to the low activity condition (18°C). This time, our schedule was designed to isolate *Imp* knockdown to development only. Developmental *Imp* knockdown caused significantly longer recovery times when compared with eGFP RNAi knockdown controls (Kruskal–Wallis *p *< 0.0001). Developmental *Imp* and adult *Imp* knockdown group recovery times were significantly different from each other with the average developmental *Imp* recovery time (15.59 s) approximately twice as long as adult *Imp* knockdown (6.738 s, Kruskall–Wallis *p *= 0.0070). There was also a much higher proportion of individuals seizing in the developmental knockdown group compared with the adult knockdown group (Fig. 2*C*). Our results suggest that *Imp* functions primarily in development to regulate seizures. In addition, developmental *Imp* knockdown (from 29 to 18°C) closely phenocopies continual knockdown (maintained at 29°C) phenotypes. Together, these results suggest that developmental *Imp* expression plays the most critical role in programming future neural function, but both developmental and adult *Imp* expression could contribute to neuronal function important for seizure regulation.

### The Imp downstream target Sdc is required for normal neuronal function and interacts with Imp molecularly and functionally

**Figure 3.**
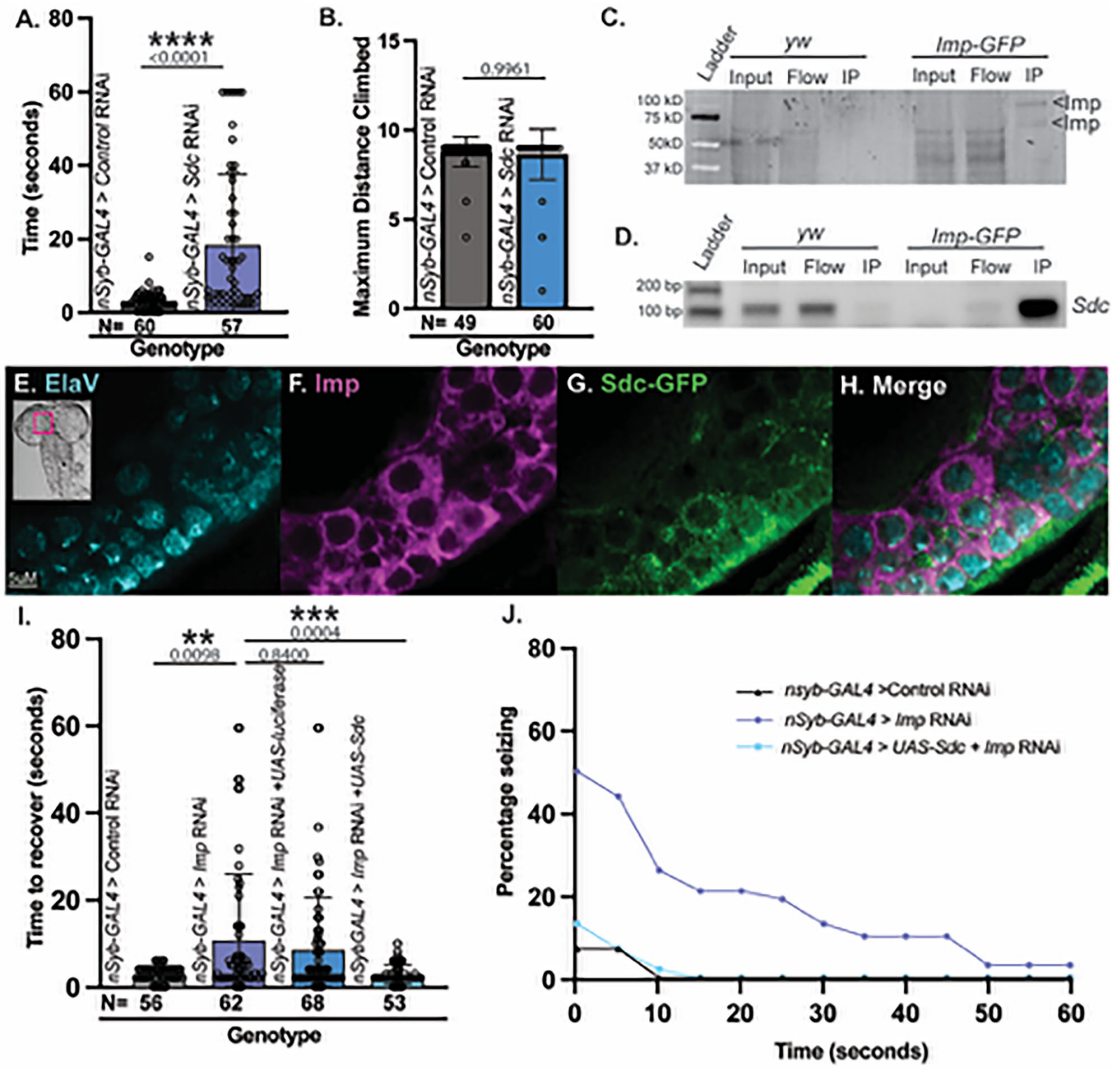
*Sdc* interacts with Imp molecularly and functionally and is required for normal neuronal function. ***A***, Average time to recover for flies exhibiting seizure behavior for neuronal (*nSyb-GAL4*) *eGFP* RNAi (control) and neuronal *Sdc* RNAi. Individual points represent each fly and whiskers represent 95% confidence intervals*. N*, number of total animals. Mann–Whitney *U* determined significance (*p* < 0.05, ***p < 0.01, ***p < 0.001,****p < 0.0001, ns = *p* > 0.05). ***B***, Maximum distance climbed (cm) in 30 s for *eGFP* RNAi control and *Sdc* RNAi. Mann–Whitney *U* significance (*p < 0.05, ***p < 0.01, ***p < 0.001,****p < 0.0001, ns = *p* > 0.05). ***C***, RNA immunoprecipitation of Imp-GFP protein from control (*yw*) and *Imp-GFP* animals using nanobody GFP agarose. Imp is immunoprecipitated specifically in *Imp-GFP* animals. ***D***, PCR analysis of Imp-GFP RNA immunoprecipitation using primers specific to *Sdc* in control (*yw*) and *Imp-GFP* animals. *Sdc* product is amplified in *Imp-GFP* RIP but not in control RIP (*yw*). ***E***–***H***, Confocal images of a single slice through the central brain of a third-instar larva stained with ElaV (neurons, cyan), Imp (magenta), and endogenous Sdc-GFP (green). Imp and Sdc proteins are both present in neurons. ***I***, Average time to recover after vortexing for flies exhibiting seizure behavior for neuronal expression (*nSyb-GAL4*) for *eGFP* RNAi (control), *Imp* RNAi, *Imp* RNAi + *UAS-luciferase* (dilution effect control), and *Imp* RNAi + *UAS-Sdc* cDNA. Points represent individual flies, whiskers represent 95% confidence intervals, and bar heights equal the mean. *p*-values are reported above each bar. *N*, number of total animals. Kruskal–Wallis determined significance between all conditions, relevant comparisons reported (*p* < 0.05, ***p < 0.01, ***p < 0.001,****p < 0.0001, ns = *p* > 0.05). ***J***, Percentage of flies seizing presented in (***E***) observed at 5 s intervals across the entire 60 s trial. Expression of Sdc cDNA in Imp knockdown animals rescues seizure deficits.

Since Imp is an RNA-binding protein, Imp's downstream target genes must be identified to molecularly understand Imp's control of seizures. Samuels et al. (2020) isolated mRNAs that bound to the Imp protein in third-instar larval brains. We leveraged this list of potential Imp mRNA targets to identify relevant candidates in seizure behavior using RNAi and vortex assays. *Sdc* knockdown was particularly interesting because it had a significantly longer recovery time compared with controls (average recovery time 18.16 s, Fig. 3*A*, Mann–Whitney *U p *< 0.0001) and exhibited robust tonic–clonic episodes after vortexing (Movie 5), suggesting that *Sdc* also is required to properly regulate seizure behavior.

**Movie 1.** Normal baseline recovery from vortexing in neural stem cell knockdown in control animals (eGFP RNAi). [View online]

**Movie 2.** Slight impairment but relatively quick recovery from vortexing for animals when *Imp* is knocked down in neural stem cells. [View online]

**Movie 3.** Normal baseline recovery from vortexing in pan-neuronal knockdown in control animals (eGFP RNAi). [View online]

**Movie 4.** Impairment in recovery from vortexing for animals with *Imp* knocked down pan-neuronally, with visible tonic–clonic episodes. [View online]

**Movie 5.** Impairment in recovery from vortexing for animals with *Sdc* knocked down pan-neuronally, with visible tonic–clonic episodes. [View online]

**Movie 6.** Recovery from vortexing in animals with *Sdc* cDNA expression in *Imp* pan-neuronal knockdown background, with fewer seizing animals and relatively quick recovery. [View online]

*Sdc* encodes a heparan sulfate proteoglycan that regulates midline axon guidance during development (Johnson et al., 2006) and the development of the neuromuscular junction (Nguyen et al., 2016), indicating that it could also function in gross motor behavior. However, the strongest candidate for downstream target analysis would cause seizure-specific deficiencies, but not gross motor deficits. To ensure we chose a strong candidate and ruled out broad motor defects, we performed forced-climbing assays on *Sdc* neuronal knockdown animals. Like *Imp*, *Sdc* knockdown did not cause any gross motor defects (Fig. 3*B*, Mann–Whitney *U p* = 0.9961). Therefore, *Sdc* is a strong candidate for seizure regulation. If *Sdc* mRNA is a target of Imp, Imp protein should bind *Sdc* mRNA. We performed RNA immunoprecipitation (RIP) in third-instar larval brains using Imp-GFP animals and anti-GFP agarose to validate that Imp binds to *Sdc* mRNA. Western analysis of RIP samples showed Imp-GFP is sufficiently immunoprecipitated (Fig. 3*C*). In addition, we found a signal for *Sdc* in Imp-GFP pulldowns, but not when using a control fly line that does not contain GFP (Fig. 3*D*, *yw*). These data suggest a strong and specific physical interaction between Imp and *Sdc* mRNA. Next, we identified whether Imp and Sdc were coexpressed in neurons in the brain. We immunostained third-instar larval brains for Imp, Sdc (using an endogenously tagged Sdc-GFP), and Elav (to mark neurons). We found that Imp and Sdc coexpress in central brain neurons (Fig. 3*E*–*H*). Coexpression of proteins in neurons supports a relevant functional interaction, and our data indicate that together, Imp and Sdc regulate neuronal function.

Physical interaction between Imp protein and *Sdc* mRNA combined with coexpression in relevant cell types is highly suggestive of a functional relationship. However, to conclude that the observed interaction is biologically relevant to our phenotype of interest, we tested whether Sdc overexpression could rescue *Imp*-induced seizures. We quantified phenotypic rescue in flies expressing *Sdc* cDNA while simultaneously knocking down *Imp* in neurons to identify such relevant interactions. *Sdc* cDNA expression significantly reduced the time needed to recover from vortexing in *Imp* knockdown animals (average time to recover, 2.83 s) compared with *Imp* knockdown alone (average time to recover, 10.79 s; Kruskal–Wallis *p *= 0.0004; Fig. 3*I*). In addition, the percentage of flies seizing was reduced to near control levels (Fig. 3*J*), and tonic–clonic episodes were nearly absent (Movie 6). Our data indicate complete rescue of seizure deficits and strongly suggest that Imp regulates *Sdc* mRNA in the context of neuronal function and seizure behavior.

## Discussion

Identifying genes that contribute to seizure disorders is an important first step in understanding how they arise and can best be treated. In this study, we demonstrate that Imp and its downstream target *Sdc* are required in neurons for seizure behavior regulation. Our study is one of few studies that has linked developmental Imp to adult behaviors (Hamid et al., 2024) and highlights the critical role of both Imp and Sdc in postmitotic neuronal development. We can now tease apart the critical developmental pathways through which Imp regulates proper neuronal function.

To identify the nature of how seizures are caused, it is important to investigate the contribution of individual cell types during development. Imp function in neural stem cells is consequential to many neurodevelopmental outcomes, but not seizure phenotypes. Interestingly, our results suggest that *Imp* expression in postmitotic neurons is most critical to prevent seizures; loss of *Imp* in neural stem cells only has no significant effect on adult neuronal function. Because little is known about the role of Imp in neurons, how *Imp* regulates development and function in the context of the terminally fated cell is an open question. We can, however, gain insight from our findings. Imp binds *Sdc* mRNA, and *Sdc* phenocopies *Imp* seizure phenotypes. These data suggest that *Imp* disruption directly impacts *Sdc*-specific phenotypes to cause seizures. Sdc is a conserved transmembrane heparan sulfate proteoglycan (Spring et al., 1994) that is secreted from midline cells of the fly central nervous system and binds products that mediate axon guidance (Nguyen et al., 2016). *Sdc* also promotes synapse growth at the neuromuscular junction (Johnson et al., 2006). Given that loss of both *Imp* and *Sdc* cause seizure phenotypes, we posit that *Imp* expression is required for proper neuronal growth because it regulates *Sdc* expression. Modifications in neural structure, particularly dendritic length and/or branching pattern, can change its firing pattern (van Elburg and van Ooyen, 2010). A vertebrate paralog of *Sdc* (Syndecan-2) promotes the formation of dendritic spines, and so would be interesting to test Syndecan-2's role in neuronal activity and seizure phenotypes. *Sdc*'s role at the neuromuscular junction is postsynaptic (Nguyen et al., 2016), so it is possible that Imp influences dendrite growth required for proper neuronal function via *Sdc* expression levels. Alternatively, loss of *Imp* and/or *Sdc* in postmitotic neurons could disrupt cell survival and induce loss of neurons. Neuronal loss has been linked to asynchronization and tonic depolarization leading to seizures (Chauhan et al., 2022). Whether loss of *Imp* causes supernumerary neuron cell death that leads to seizures and whether *Sdc* overexpression could rescue this phenotype are intriguing and an essential future study.

Though Imp has been studied extensively in a developmental context, our data suggest that *Imp* plays a potential role in adult neuronal maintenance and function. This study sought to determine when *Imp* function was required for seizure regulation. Our results strongly suggest that developmental Imp has the most influence on neuronal function later in life. However, flies with reduced Imp only in adulthood still experienced a marked increase in seizures, though not statistically significant, while loss of developmental Imp caused more severe seizures with a significant increase in duration and a higher likelihood of occurrence. Our results are in line with what is known about Imp being a key factor in early brain development that influences adult brain circuitry (Munroe and Doe, 2023) and behavior (Hamid et al., 2024). Beyond development, RNA-binding proteins play roles in cell migration, maturation, and synaptic integration in mature brain cells (Chan et al., 2022). Imp, therefore, could be a player in neuronal maintenance that suppresses seizures. Identifying changes in neuronal circuitry during development as well as excitation and inhibition imbalances in animals with only adult Imp loss will be the first important step in disentangling the functional role of Imp.

The seizures observed in both *Imp* and *Sdc* neuronal knockdown animals were relatively short but conspicuous and stereotyped. Clear tonic–clonic episodes were observed in all trials. Previous *Drosophila* epilepsy studies suggest that time to recover determines the severity of seizures (Fischer et al., 2023). However, seizure length is a superficial way to characterize seizure phenotype. The *Imp*-related spasms observed in our study are similar to the seizure characteristics of other seizure models, but they lack an initial paralysis stage, resulting in a shorter recovery time. Though they have a relatively short recovery time compared with age-matched classic mutants such *para^bss^* (average, 491 s; Reynolds, 2018), *eas* (average, 140 s; Zhang et al., 2002), and *sda* (average, 37 s; Zhang et al., 2002), they have distinctive initial spasms, short paralysis bouts, and recovery seizure stages before their recovery. Several factors may contribute to the relatively less severe phenotypes that we observe. Importantly, we are only knocking Imp down in a single cell type, whereas all described seizure models are mutants. The mentioned seizure model genes are expressed in multiple cell types in addition to neurons that may contribute to seizure severity including glia, sensory neurons, motor neurons, and muscles (Li et al., 2022). Imp is also expressed in all of these cell types, which could explain the lack of paralysis and normal climbing observed in our model compared with established bang-sensitive mutants that have general motor performance defects (Reynolds, 2018). An additional consideration is that these knockdowns have only a single copy of both the GAL4 driver and the RNAi. In established mutant models, stronger phenotypes are observed in homozygous animals (Song and Tanouye, 2006). Because *Imp* knockdown is partial, downstream regulation of *Sdc* is likely diluted. We would not expect our phenotype to be as severe as observed in null models. Future studies will investigate how seizure severity changes with loss of Imp in different cell types and with ubiquitous loss of Imp. Importantly, we show that Imp knockdown in neurons alone is sufficient to generate seizures without any other behavioral defects. We can therefore leverage this seizure-specific phenotype to probe mechanisms unambiguous to seizure activity.

We provided evidence for a functionally relevant interaction between *Imp* and *Sdc* in seizure regulation, but a limitation of this study is that we have yet to identify the molecular mechanism by which *Imp* mediates Sdc expression. RNA-binding proteins modulate gene expression by stabilizing downstream mRNAs, modifying transcription and translation, and transporting mRNAs to different parts of the cell (Prashad and Gopal, 2021; Parra and Johnston, 2022). Multiple studies have identified that Imp controls neurodevelopmental processes by regulating downstream target mRNA expression (Medioni et al., 2014; Liu et al., 2015; Yang et al., 2017; Samuels et al., 2020; Guan et al., 2024), but the mechanisms regarding *Sdc* mRNA have not been explored. Because *Sdc* cDNA overexpression rescues seizure phenotypes in *Imp* knockdown, it is possible that Imp positively regulates *Sdc* mRNA or protein levels. Molecular assessment of how the loss of Imp changes *Sdc* mRNA and protein levels, as well as any changes in *Sdc* mRNA localization, will elucidate the mechanism by which Imp regulates *Sdc* and how this interaction relates to seizure regulation.

Like many RNA-binding proteins, Imp has numerous downstream targets. In addition to *Sdc*, some Imp targets such as *Para* (Royden et al., 1987; Pavlidis et al., 1994; Glasscock and Tanouye, 2005; Tao et al., 2011; Tapia et al., 2021) and *Shaker* (Papazian et al., 1987; Kuebler et al., 2001; Song and Tanouye, 2006) have previously been characterized for their role in seizure biology. Samuels et al. (2020) identified an additional 37 Imp targets in larval brains, so other pathways could be involved in seizure control. Additionally, other cell types could affect seizure regulation. Glia, for example, provide critical support for extracellular matrix (ECM) remodeling and perineuronal net integrity (Meyer et al., 2014; Kim et al., 2016; Nguyen et al., 2020; Tewari et al., 2022) both of which influence seizure occurrence (Dityatev and Fellin, 2008). Given that heparan sulfate proteoglycans such as *Sdc* are ECM proteins (Spring et al., 1994; Sarrazin et al., 2011), it will be important to investigate the role of both *Imp* and *Sdc* in glia during development and in adult brain function.

In conclusion, we show a role for the RNA-binding protein Imp in postmitotic neuronal regulation of brain function, where the loss of neuronal Imp expression causes seizure behaviors. Additionally, we provide support for a functionally relevant interaction between Imp and Sdc in seizure behavior. Our work contributes to a growing body of literature highlighting the role of RNA-binding proteins as critical regulators of brain function and opens many new exciting questions about how *Imp* and other RNA-binding proteins could regulate brain development and function through their role in postmitotic neurons.

## Synthesis

Reviewing Editor: Catherine Schevon, Columbia University Decisions are customarily a result of the Reviewing Editor and the peer reviewers coming together and discussing their recommendations until a consensus is reached. When revisions are invited, a fact-based synthesis statement explaining their decision and outlining what is needed to prepare a revision will be listed below. The following reviewer(s) agreed to reveal their identity: Richard Baines, Tristan Sands.

The revised manuscript is improved. A few minor changes are requested to improve clarity and accuracy.

1. The terms 'development' and 'mature' are not well explained in the section using temperature to manipulate nsyb GAL4. The methods section should make clear which stages of the life cycle were manipulated. Does 'developmental' include embryo, larva and pupa in addition to young adult, whilst mature is restricted to just adult flies?

2. The discussion states that sda adults seize for ~20 secs. This is not fully accurate. Zhang et al states that the initial paralysis takes ~20sec. However, this is not full recovery, and adults go on to show post-paralysis hyperactivity. This study reports a 50% full recovery rate takes ~ 37 sec and their figure (fig 1) shows 100% recovery of all flies takes ~ 60 secs. Note, eas and para(bss) tested at the same time in this same study exhibit 50% recovery times or 140 and 150 sec, respectively.

3. The contention that imp KD seizures are short because only one cell type is being manipulated is a little disingenuous. nSyb expresses in all neurons. Thus, whilst glia may contribute to seizures, it seems that all neurons should produce a near maximal effect, in most instances. Of course, this could be tested using a pan-cellular GAL4 such as an actin driver (or similar). For this argument to hold, the authors would need to demonstrate that established seizure mutations are expressed more widely than neurons.

4. The inability to demonstrate a direct effect on syndecan remains a weakness of the manuscript. This is fine but then the title needs to be altered to more accurately reflect what has been demonstrated. This should also be explicitly described as a study limitation in discussion.

## Author Response

The revised manuscript is improved. A few minor changes are requested to improve clarity and accuracy.

We thank the reviewers for the helpful comments that improve clarity of our manuscript. We have addressed each comment individually below.

1. The terms 'development' and 'mature' are not well explained in the section using temperature to manipulate nsyb GAL4. The methods section should make clear which stages of the life cycle were manipulated. Does 'developmental' include embryo, larva and pupa in addition to young adult, whilst mature is restricted to just adult flies? We updated our methods section to include the developmental stages and made the sentence indicating time of adult collection more transparent.

2. The discussion states that sda adults seize for ~20 secs. This is not fully accurate. Zhang et al states that the initial paralysis takes ~20sec. However, this is not full recovery, and adults go on to show post-paralysis hyperactivity. This study reports a 50% full recovery rate takes ~ 37 sec and their figure (fig 1) shows 100% recovery of all flies takes ~ 60 secs. Note, eas and para(bss) tested at the same time in this same study exhibit 50% recovery times or 140 and 150 sec, respectively.

We updated the discussion section with the full recovery time, not just recovery after paralysis. We also elaborated on paralysis states in our model vs the established seizure models as well as our interpretation.

3. The contention that imp KD seizures are short because only one cell type is being manipulated is a little disingenuous. nSyb expresses in all neurons. Thus, whilst glia may contribute to seizures, it seems that all neurons should produce a near maximal effect, in most instances. Of course, this could be tested using a pan-cellular GAL4 such as an actin driver (or similar). For this argument to hold, the authors would need to demonstrate that established seizure mutations are expressed more widely than neurons.

We included expression information in our discussion.

4. The inability to demonstrate a direct effect on syndecan remains a weakness of the manuscript. This is fine but then the title needs to be altered to more accurately reflect what has been demonstrated. This should also be explicitly described as a study limitation in discussion.

We have changed the wording of the title to exclude "induces" as requested by the editor as well as added an explicit statement in the discussion siting the lack of molecular mechanism as a study limitation.
