# Supplementary figures and images for: Loss of Neuronal Imp Contributes to Seizure Behavior through Syndecan Function
Source: eNeuro. 2025 May 2;12(5):ENEURO.0545-24.2025. doi: 10.1523/ENEURO.0545-24.2025 (PMC12052222; doi:10.1523/ENEURO.0545-24.2025)

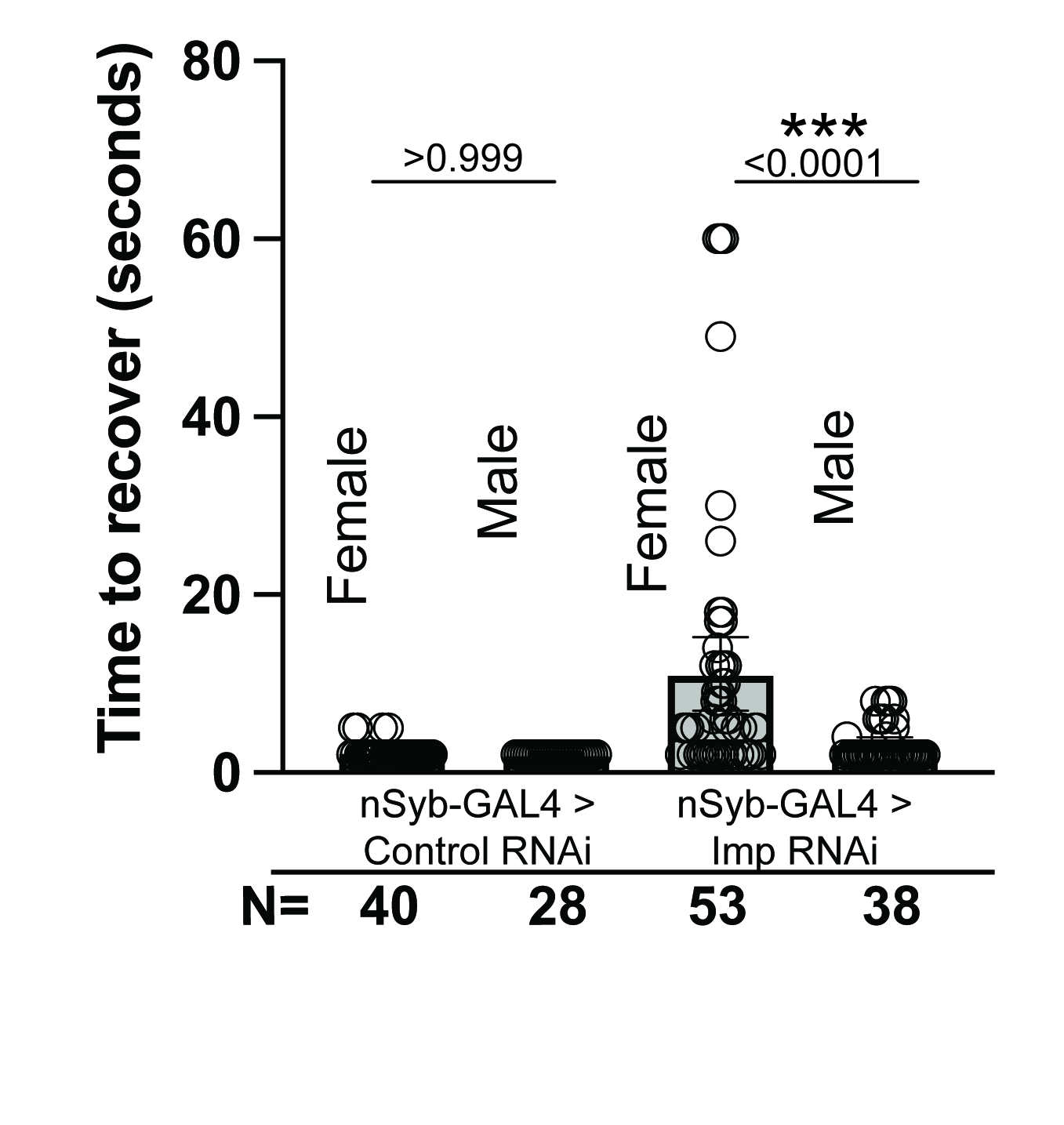

Supplement: Figure 1-1 — Imp knockdown has a stronger effect on females. Seizure behavior reported as average time to recover after vortexing for eGFP RNAi (VALIUM22-EGFP.shRNAI.1) Control and Imp RNAi (TRIP.HMC03794) expressed using pan-neuronal driver neuronal synaptobrevin-GAL4 (nSyb-GAL4). Individual points represent each fly, whiskers represent 95% confidence intervals, and bar heights equal the mean. Kruskal-Wallis test determined significance between all conditions. Relevant comparisons reported (p<0.05, ***p<0.01, ***p<0.001,****p<0.0001, ns=p>0.05). N= number of total animals. Download Figure 1-1, TIF file. [file eneuro-12-ENEURO.0545-24.2025-s002.tif]
